# Aspects to consider in causality assessment of safety signals: broadening the thought process

**DOI:** 10.3389/fdsfr.2023.1193413

**Published:** 2023-05-25

**Authors:** Tarek A. Hammad, Salman Afsar, Laura B. McAvoy, Hervé Le Louet

**Affiliations:** ^1^ Head of Medical Safety, Marketed Products and PV Policy Intelligence Strategy, Global Patient Safety Evaluation, Takeda Pharmaceuticals Inc., Cambridge, United States; ^2^ Medical Safety Assessment Physician and Program Lead, Worldwide Patient Safety, Bristol Myers Squibb, Princeton, United States; ^3^ PV Science, Worldwide Patient Safety, Bristol Myers Squibb, Princeton, United States; ^4^ Head of PV Policy Intelligence Strategy, Global Patient Safety Evaluation, Takeda Pharmaceuticals Inc., Cambridge, United States; ^5^ President of the Council for International Organizations of Medical Sciences (CIOMS), Geneva, Switzerland

**Keywords:** drug safety, causality assessment, evidence-based medicine, structured framework for causality, holistic safety signal assessment approach, pharmacovigilance

## Abstract

In the field of drug safety, causality assessment aims to determine the level of plausibility of the relationship between an adverse event and exposure to a particular product. It is after the causality assessment process that we will be able to point out a product adverse reaction. While regulators often require pharmaceutical companies to use a structured approach for assessing the causality of their products, the available methods are challenged by a number of procedural differences, even when drawing from the same domain of elements. To mitigate these inconsistencies, as well as the additional challenges associated with incomplete information and differences in the application of clinical judgments at the individual case level, this paper proposes a holistic framework for causality assessment that utilizes a combination of expert judgment/global introspection, evidence-based medicine, and probabilistic method. The goal of the presented approach is to provide a guide of clues to causality reminding medical safety assessors to seek and examine all available streams of evidence in totality and to assess this evidence in a qualitative, structured way.

## 1 Introduction

Safety signals are defined as “information arising from one or multiple sources, including observations and experiments, which suggests a new potentially causal association, or a new aspect of a known association between an intervention and an event or set of related events, either adverse or beneficial, that is judged to be of sufficient likelihood to justify verificatory action^1^.” Once a signal is identified, further assessment ensues to identify adverse reactions. In accordance with the ICH-E2A^2^, the definition of an adverse reaction implies at least a reasonable possibility of a causal relationship between a suspected medicinal product and an adverse event (AE). Therefore, the safety signal assessment process evaluates the potential causality association between a given product and a particular adverse event. It is an evaluation of the likelihood that a particular treatment is the cause of an observed adverse event (Pande, 2018).

Regulators often require pharmaceutical sponsors to use a structured approach for causality assessment (as per EMA guidance^3^, for example). However, the available methods for causality assessment focus mostly on individual case reports and are challenged by differences in the implicit use of clinical definitions of adverse events, inconsistent use of terminology, variation in the categorization of levels of causality, and incomplete information from case reports coupled with vast differences in the application of clinical judgments (Agbabiaka et al., 2008). Interestingly, most available methods draw more or less from the same domain of elements but vary in how they utilize these elements. The causality assessment methods might be roughly grouped into three broad categories: expert judgment/global introspection (e.g., WHO-UMC system), algorithm-based that can be divided into generalist or organ class specific algorithms (some associated with scoring, for example, Naranjo scale and French causality assessment) and probabilistic (e.g., Bayesian) methods. No particular method is universally accepted, though the expert judgment/global introspective approach is used most commonly, as algorithm-based and probabilistic methods have been shown to be difficult to implement reliably in practice (Agbabiaka et al., 2008).

While an expert judgment approach is relatively simple and quick, poor reproducibility and low intra- and inter-rater agreement between similarly trained individuals have been well-documented (Comfort et al., 2018). In fact, comparability between assessors was found to be “fair” or less for the causality assessment methods examined in some studies (Davies et al., 2011 ^4^; Arimone et al., 2005; Arimone et al., 2007). This is highly dependent on the quality of data, so good expert judgment performed by a highly trained healthcare professional using many streams of evidence is better than a poorly designed algorithm.

Most causality assessment methods rely, at least partially, on assessing causality per individual cases (Sartori et al., 2022), which might enable the detection of novel new and unexpected signals and adverse reactions. However, the challenge of causality assessment in the face of limited or incomplete information is particularly evident in the post-marketing setting with spontaneously reported cases of adverse events. Given these familiar challenges at the case level, the role of additional sources or streams of evidence in the process of safety signal assessment should be explored by combining individual case-level and population-based level assessments when investigating safety signals. This paper describes an approach for causality assessment in drug safety that combines several available tools in the armamentarium of evidence-based medicine, which facilitates communicating the reasoning behind the assessment.

## 2 Outline of the proposed approach

The approach proposed in this paper leverages a combination of methods and focuses on simplicity and the need for flexibility, consistency, and transparency in the assessment process.

The aspects considered by this approach reflect principal elements of a framework that supports a causality assessment thought process and do not represent a comprehensive guide to causality assessment. The idea is to provide a guide of clues to causality that works as a reminder for medical safety assessors to seek and examine all available pieces of evidence, as applicable, in order to frame their thinking as well as help them assess the totality of the evidence in a qualitative, structured way. In general, two distinct aspects are often conflated and should be performed independently (Table 1): A) characterizing the diagnosis and definition of the clinical condition (the AE) and B) assessing the evidence for causality (finding clues to causality). The idea is to parse out how strongly the safety assessor feels about the clinical characterization of the AE reported in temporal proximity to the product intake from how much evidence there is to support causal association.

**TABLE 1:** Aspects to consider in safety signals causality assessment, questions to ask, and general principles governing the thought process.

Aspect to consider	General principles/elements to assess
A) Characterizing the diagnosis and definition of the clinical condition (the AE)	
1) What is the risk under evaluation?	Be as specific as possible based on observations from the source of the safety signal rather than theoretical association driven by potential mechanisms of action
2) For complex medical concepts, how to establish a case definition and facilitate differential diagnosis?	Be as specific as possible with consideration given to cases’ demographic information and the clinical course of the event. Specify the clinical parameters that would help in making a distinction between clinical conditions that might have some elements in common
3) What is the diagnostic certainty of the defined adverse event among the reported cases?	Try to locate and utilize a validated approach with known positive predictive value
B) Assessing the evidence for causality (finding clues to causality)	
1) Individual patient-level evidence	• Positive re-challenge
	• Positive de-challenge
	• Known biologically plausible mechanism
	• Plausible time-to-onset
	• Possible confounders/alternative explanation(s)
	• Recognized class effect
2) Aggregate/population-level evidence	• Study-driven findings
	• Case clustering
	• Supporting findings in non-clinical investigations, for example, laboratory or animal models
	• Observed-to-expected rates
	• Probability calculations of chance finding
	• Hierarchy of evidence considerations
3) Overall judgment on causality	Overall judgment on causality should consider evidence from both individual and aggregate levels, as available. However, not every source of evidence has equal weight

### 2.1 Characterizing the diagnosis and definition of the clinical condition (the AE)

Properly understanding and contextualizing the mostly rare post-marketing events (e.g., toxic epidermal necrolysis, thrombotic thrombocytopenic purpura, or hemophagocytic lymphohistiocytosis), as well as delineating true cases from anecdotal reports, is important. The first step in the proposed approach is to examine the available clinical information to assess the diagnostic accuracy of the clinical condition to the extent possible from the information being reported by answering several important questions, which are discussed in the following sections.

#### 2.1.1 Question 1: what is the risk under evaluation?

When addressing this question, it is important to be as specific as possible based on observations from the source of the safety signal, not based on theoretical association driven by the potential mechanism of action of the drug in question. Optimally, the characterization of the AE should be based on a consensus expert guideline for the diagnosis and management of the clinical condition. Guidelines or validated reference articles for diagnostic criteria might be unavailable or not sufficiently accredited. However, when available, the diagnostic criteria must be cautiously applied. For example, the original RegiSCAR score was published in a letter (Kardaun et al., 2007) in which a two-stage process for collecting cases of drug rash with eosinophilia and systemic symptoms (DRESS) for further study is discussed: 1) collection of potential cases of DRESS (“inclusion criteria”) followed by 2) confirmation of the diagnosis (“validation criteria”). The inclusion criteria are meant to define a population of potential cases suitable for further study and evaluation, while the validation criteria are meant to establish a diagnosis (Kardaun et al., 2013; Kardaun et al., 2014). However, a continuous medical education article (Husain et al., 2013) made a mistake and presented the RegiSCAR inclusion criteria rather than the validation criteria (Kardaun et al., 2014). Ideally, relevant guidelines can be located, as applicable, and the original papers that developed the criteria should be reviewed to understand the clinical context for their development and application.

Additionally, it is important to not conflate the MedDRA Standardized or Customized Query (SMQ/CMQ) used as the search criteria with the eventual case definition of the AE. When searching the safety databases, the SMQ/CMQ used is often too broad and includes medical concepts intended to cast a wide net to make sure all potential cases are captured. The idea is that once the assessor has searched for and captured all potentially relevant cases, medical judgment is required to identify and finalize the specific AE that will be investigated under the safety signal.

#### 2.1.2 Question 2: how to establish a case definition and facilitate differential diagnosis for complex medical concepts?

An established case definition of the AE should be as specific as possible, particularly for those clinical conditions with complex medical concepts as shown in the DRESS example in the previous question. Additionally, this case definition should clearly identify clinical parameters that help separate the cases of interest from other case reports in the safety database that might have some elements in common to facilitate the differential diagnosis. In the process, a list of the other clinical conditions in a differential diagnosis (DD) setting should be clearly identified. Subsequently, these clinical parameters should be reviewed in the case histories, as available, to help in supporting the diagnosis among the reported cases and adding clarity to the nature of the AE of interest.

Additionally, establishing a case definition may include aspects concerning a particular population of interest (e.g., events reported in pediatrics, events occurring in patients with a minimum number of treatment cycles/doses received, etc.) or an identified detail related to event progression and/or management (e.g., events reported as ongoing vs. resolved following a certain treatment regimen specific to the clinical condition, events reported following a pre-defined latency period, etc.). This can be very helpful, especially when dealing with post-marketing case reports that tend to provide a paucity of information, and this piece of additional information might prove to be integral for the search strategy. For instance, considering management strategies such as steroids and immunosuppression can facilitate diagnostic confirmation and identification of some AEs, such as autoimmune hepatitis.

As an example, the initial clinical picture can be similar for immune thrombocytopenic purpura (ITP) and thrombotic thrombocytopenic purpura (TTP). Considering the overlap in clinical signs and symptoms, it is of utmost importance that a clear case definition facilitating the identification of cases is identified. Compared to ITP, TTP has a poor prognosis (without treatment, the mortality rate for TTP is 90% within 10 days of disease onset)^5^. Therefore, identification and safety database retrieval of case reports would be challenging without utilizing the clinical course, therapeutic intervention, and specific case definition of TTP, as detailed in Table 2. This would be attainable to the extent that the information can be found in the case report or obtained from requesting targeted follow-up of patients.

**TABLE 2:** Example of parameters that might aid in the differential diagnosis between ITP and TTP cases.

Parameter	Immune thrombocytopenia purpura (ITP)	Thrombotic thrombocytopenic purpura (TTP)
Pathogenesis	Anti-platelet antibodies	Endothelial defect ADAMTS13 Ab
RBC	Normal level (NL)	Schistocytes
PT-(INR)	NL	NL/slightly increased
PTT	NL	NL/slightly increased
Fibrinogen	NL	NL
Fibrin monomers	NL	Slightly increased
Fibrin degradation	NL	Slightly increased
D-Dimers	NL	Slightly increased
Therapy	- Steroids	- Plasma exchange
	- IVIG	- Vincristine
	- Splenectomy	- Rituximab

#### 2.1.3 Question 3: what is the diagnostic certainty of the defined AE among the reported cases?

In order to assess the diagnostic certainty with the highest level of plausibility of the defined AE, a validated approach with known positive predictive value should be identified and located as available, for example, the ADAMTS13 deficiency for TTP and the Yamaguchi criteria for adult-onset Still’s disease (AOSD). The original papers that reported the validation effort should be read closely to understand it better and appreciate the implication of the extent of the validation on the performance of these approaches within the context of the information available for the reported cases. For example, the ADAMTS13 activity assay had a positive predictive value for TTP of 91% and a negative predictive value of 100% using a 20% activity level cutoff with appropriate exclusions for interfering conditions. Therefore, not any deficiency level would be considered diagnostic (Barrows and Teruya, 2014). Another example is the AOSE, which is a diagnosis of exclusion. The Yamaguchi criteria diagnostic of AOSE assume the absence of infections, malignancies, and other rheumatic disease (Yamaguchi et al., 1992). However, some case reports might not assert that investigations have been undertaken to rule out these conditions. In the process, diagnostically definitive cases, as available, should be tracked for use with sensitivity analyses.

### 2.2 Assessing the evidence for causality (finding clues to causality)

The need to consider several streams of evidence in assessing causality in drug safety is paramount, especially in the post-marketing settings with no one particular steam that can provide a definitive answer. Although there are no absolute criteria for assessing the validity of scientific evidence, a careful critical assessment can enhance the value of available evidence. Leveraging several streams of evidence also augments the overall confidence in the eventual judgment about causality. Therefore, there is no alternative, as part of the assessment, to examining all available sources of evidence and qualitatively evaluating pertinent quality attributes to the extent possible. Achievement of this goal requires more than the mere application of a list of criteria; it starts by identifying the domain of streams of evidence that can be pertinent to the assessment process.

**FIGURE 1:**
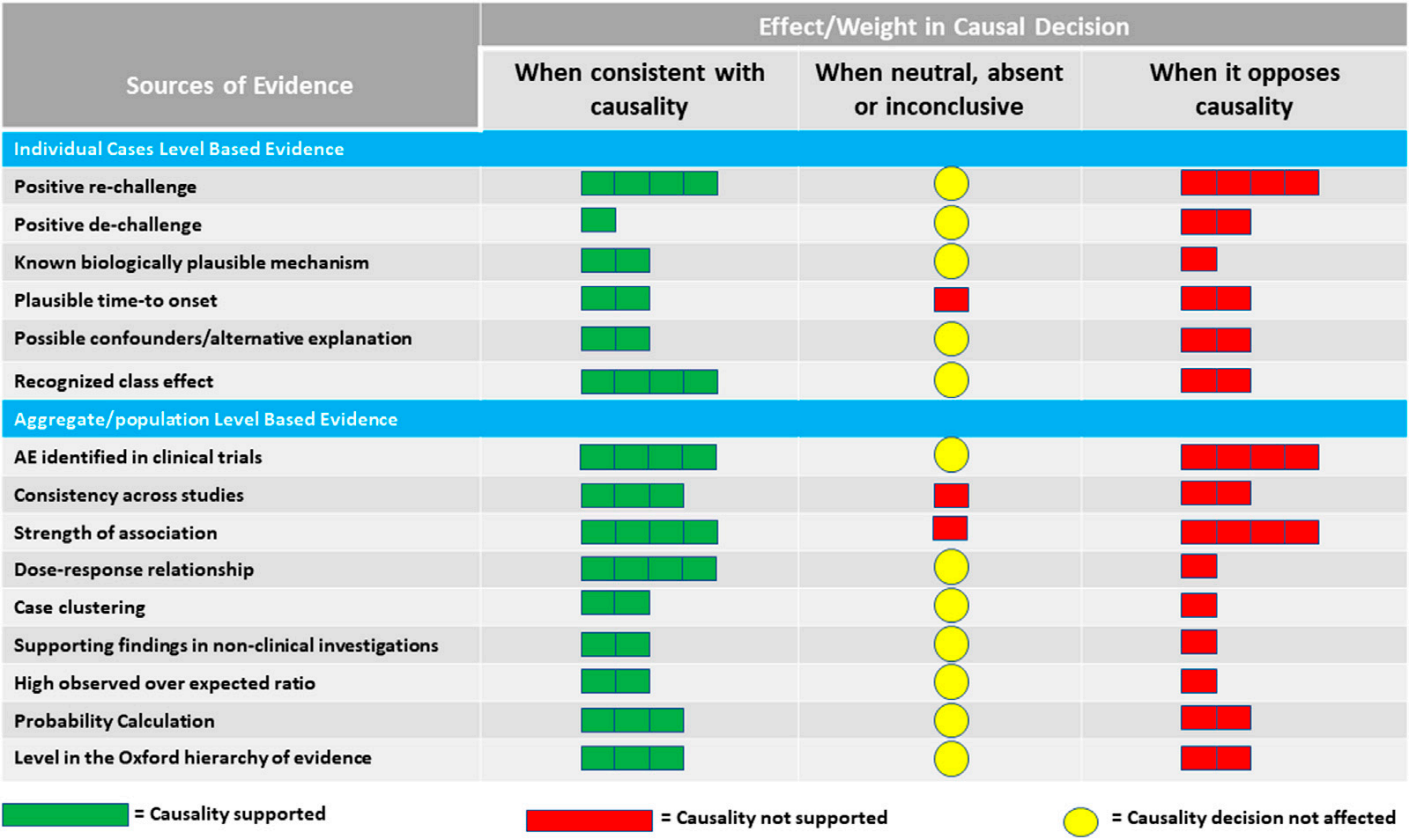
Proposed qualitative weights reflecting the effect of sources of evidence on judging causality.

Clues to causality come from elements of two sources or levels of evidence: individual patient level and aggregate/population level. The proposed elements represent a collection of aspects, some of which are present in other frameworks, such as the WHO-UMC system for standardized case causality assessment^6^ or the Bradford Hill’s Causality Criteria (Hill, 1965), as well as some additional evidence-based medicine tools. These aspects are not intended as “checklist criteria” as much as they provide a list of what to consider when making a judgment on causality. They provide a way to minimize inter- and intra-assessor variability by highlighting all the elements that need to be considered. Such an approach avoids the temptation to use causal criteria to support a preconceived notion about the causal relationship between a drug and a particular AE and instead allows assessors to focus on evaluating the potential for causal association using a well-rounded set of crucial observations. Scientific recommendations might be widely followed if they provide easy guidance, but recommendations that call for complex actions might be ignored (Hofler, 2005). To assemble the information and make an overall judgment, a simple graphical approach (Figure 1) is proposed, suggesting different qualitative weights in the decision-making for each source of evidence.

The focus of this approach is not on the assessment of causality in a particular patient but on the larger question of whether the drug is likely causing the AE in the patient population. It is important to note, however, that caveats and counterfactual arguments exist to some of the elements in the proposed approach. For example, while a strong association reported from studies may be supportive of a causal component, the absence of a strong association does not necessarily exclude causality (Hofler, 2005; Rothman and Greenland, 2005). The elements presented in the following sections provide useful information that addresses nuances that may help when using the proposed approach to investigate a particular safety signal.

#### 2.2.1 Individual patient-level evidence

At the patient level, the causality designation should be investigated based on a thorough evaluation of the case history using findings from the following elements:

##### 2.2.1.1 Positive re-challenge/positive de-challenge

Positive re-challenge is encountered when the AE occurs again, if it initially subsided with the removal of the drug, after the reintroduction of the drug in question. It represents one of the strongest types of evidence for a causal association in drug safety. However, it should be considered only for diagnostically confirmed AEs. Such a case, when not confounded, might represent a sentinel or index case reflecting a higher level of confidence in the evidence. On the other hand, positive de-challenge is observed when the AE ceases after the drug is discontinued and not readministered. It does not carry the same weight as positive re-challenge as the disappearance of the AE might be a function of its cyclical nature and not necessarily related to stopping drug intake. It is important to note that when a corrective treatment is administered, the effect of de-challenge cannot be accurately assessed.

##### 2.2.1.2 Known biologically plausible mechanism

Biological plausibility is an important but not absolute concern. An implausible explanation might be correct in some scenarios (Rothman and Greenland, 2005). We should not discount the significance of identifying a mechanism of action but need to appreciate the challenges. The potential for a novel, off-target mechanism for an adverse effect exerted by a particular drug precludes our ability to use the absence of a known mechanism to exclude causality association altogether. On the other hand, the presence of a theoretical mechanism based on laboratory or animal work might not always be supportive of causality due to the complexity of the biology of the human body. The breakthrough of therapeutic biologics will certainly be a major challenge in this regard.

##### 2.2.1.3 Plausible time to onset

A relevant time to onset of an adverse event is subject to the mechanism of action of the drug that might require a one-time exposure or a cumulative long-term exposure. One pertinent aspect is that the extent and duration of exposure should be plausible based on the known PK/PD profiles and the nature of the AE. For example, cancer might require a cumulative prolonged exposure period. However, a carcinogenesis promotor might behave differently from an initiator in terms of the time it needs to induce its effect^7^. Also, a drug with a noticeably short half-life that clears quickly from the body is not a plausible cause of an AE that occurs several weeks after exposure ends.

##### 2.2.1.4 Possible confounders/alternative explanation(s)

Confounding is a crucial aspect in assessing individual case reports during the investigation of a safety signal. Ignoring confounding can lead to flagging spurious causal associations. The confounding effect can materialize due to the presence of a risk factor for the AE of interest or due to a concomitant drug or comorbidity known to be associated with the AE. However, this should not totally preclude the potential for the drug effect on the adverse event, as this effect cannot be reliably excluded in some scenarios. It should be noted that the incidental presence of a risk factor or a potential alternative explanation might not mean a confounding effect has been exerted.

Caution should be exercised in the assessment in order to not count clinical events in the “causal chain” as confounders. For example, if we are assessing acute myocardial infarction (MI) as an adverse event, we should not count the presence of hypertension in a patient’s medical history as a confounder if the drug being investigated is known to cause hypertension. Although hypertension is a risk factor for MI, it would be in the causal chain in this particular scenario.

##### 2.2.1.5 Recognized class effect

Some drug classes are known for their causal association with certain AEs, such as statins and rhabdomyolysis. Usually, the mechanism of action of drugs in the same class is more or less the same, so it is reasonable to expect that the AE profiles can be shared among these drugs. However, some drugs might have a more pronounced effect than others in the same class. For example, cerivastatin (Baycol) had a higher reporting rate of rhabdomyolysis and was withdrawn from the market (Furberg and Pitt, 2001). However, when the mechanism of action behind the AE is not known, it becomes more difficult to exclude a particular drug in the class from the causal association.

#### 2.2.2 Aggregate/population-level evidence

At the aggregate level, the causality designation should be investigated in consideration of the following elements, as applicable.

##### 2.2.2.1 Study-driven findings

If one or more studies explore the association between the drug in question and the AE, it is important to examine aspects of the findings that might strengthen the presence of causality, for example, consistency of the association across studies, reports of a strong association, including a high magnitude of effect, and positive dose–response relationships. Information about these aspects might not always be available, but it is worth investigating if there is evidence of it in the drug’s clinical development program or in the literature. Nonetheless, this aspect has its own set of methodological limitations (Hofler, 2005; Rothman and Greenland, 2005). For dose–response, a low dose received does not exclude a causal relationship with the drug as idiosyncratic drug reactions might be dose-independent. Additionally, the nuances of the study design might have a considerable impact on the quality of the findings in terms of how the design minimizes potential residual confounding or sources of bias. For example, in a trial with a randomized withdrawal design, patients who do not tolerate the drug are usually excluded from entering the second phase of the trial and might not contribute to the overall results regarding AEs. In general, an expert opinion should be solicited to ensure the study results are of sufficient quality for use in the overall judgment on causality. This type of assessment cannot be performed easily by someone who lacks the skills and training of a scientist familiar with the subject matter and the scientific methods that were employed (Rothman and Greenland, 2005). In general, randomized clinical trials rank highly in the weight of evidence and should be treated as such when weighing the totality of the available evidence.

##### 2.2.2.2 Case clustering

Clustering of cases reported, for example, by indication, age, gender, geography, or time to event, might be helpful in understanding the nature of the safety signal and, eventually, informative to patients and healthcare providers. The assessor should look for and describe case clustering and its implications on the interpretation as it relates to the evidence for or against causality. A cluster of cases reported from one center in a clinical trial conducted in several centers should be examined with caution as it might reflect differences in training or execution, some types of protocol deviation, or a particular bias in reporting (Jo, 2020).

##### 2.2.2.3 Supporting findings from non-clinical investigations (e.g., laboratory or animal models)

The translational value of non-clinical findings depends on the chemical nature and mechanism of action of the drug being investigated. Due to unique aspects of human biology, laboratory and animal model findings can only be suggestive of a potential safety finding in humans and do not carry a high weight until the AE is observed in a clinical setting. On the other hand, the absence of findings in the non-clinical stage does not strongly support the lack of a causal relationship between the AE and the drug being investigated.

##### 2.2.2.4 Observed-to-expected ratio

Optimally, to calculate such a ratio, we should try to find the incidence of the AE in both the general population and the patient population of interest. The idea is to compare the observed rate of the AE (e.g., incidence in a trial or, as a rough proxy, reporting rate (RR) in post-marketing) to the background incidence rate in the general population and in the patient population/subpopulation, as applicable. Subpopulations may be identified based on stratification, for example, by indication, age, and gender. The concept of utilizing rates of “anticipated” AEs in the patient population of interest is supported by the most recent FDA safety assessment guidance^8^, albeit in the context of IND safety reporting.

Comparing an RR for an adverse event of interest and incidence rates for the same event from other independent data sources, such as estimates in the literature, is a crude exercise for several reasons. First, the numerator used to generate the reporting rate may not be accurate given the potential underreporting of adverse events. Second, the denominator (population exposure to the drug) is an estimate based mostly on sales data, as it is not always possible to get the exact number of exposed patients. Therefore, the RR does not represent the incidence of the AE but is a rough proxy of the rate of encountering and reporting the AE among exposed patients. To this end, the RR should be interpreted with caution, and relevant sensitivity analyses might be warranted. The limitations notwithstanding, the exercise can sometimes be useful in some scenarios to indicate whether the occurrence of an event is out of range for what might be typically expected. For example, if the observed reporting rate of an AE among patients exposed to a particular drug is meaningfully larger than the expected one, it might strengthen the case for a causal association between the drug and the AE. On the other hand, some factors can lead to stimulated reporting, like media attention or litigation-driven reporting. This can sometimes lead to a particular drug in a class appearing to have a higher RR than other members of the class. Such factors should be investigated closely before using the observed-to-expected ratio approach.

##### 2.2.2.5 Probability calculation

Another stream of evidence requires calculating the probability of encountering the observed number of the AE cases by chance among exposed patients, given the background incidence rate of the AE and the extent of exposure to the drug. The primary approach might be to use the diagnostically definitive observed AE cases with a sensitivity analysis that includes all observed cases as well as all observed cases multiplied by up to 10 (to account for potential under-reporting). The key is to use the appropriate probability calculations based on the nature of the unit of the denominator (number of patients *versus* number of patient years). Online resources are available to perform such calculations quickly (use a binomial probability calculator if the denominator is the number of patients^9^ and a Poisson distribution calculator if the denominator is the number of patient-years^10^).

##### 2.2.2.6 Hierarchy of evidence

The Oxford Center for evidence-based medicine provides several levels for the hierarchy of evidence for harm (Table 3). This tool can be used in the overall assessment of the level of evidence available while investigating a particular safety signal^11^.

**TABLE 3:** Oxford Center for Evidence-based Medicine 2011 levels of evidence for harm.

	Step 1 (Level 1)^a^	Step 2 (Level 2)^a^	Step 3 (Level 3)^a^	Step 4 (Level 4)^a^	Step 5 (Level 5)^a^
COMMON harms	Systematic review of randomized trials, systematic review of nested case–control studies	Individual randomized trial, or (exceptionally) observational study with dramatic effect	Non-randomized controlled cohort/follow-up study (post-marketing surveillance) provided there are sufficient numbers to rule out a common harm. (For long-term harms, the duration of follow-up must be sufficient.)^b^	Case–series, case–control, or historically controlled studies^b^	Mechanism-based reasoning
	*n*-of-1 trial with the presenting patient or observational study with dramatic effect				
RARE harms	Systematic review of randomized trials or *n*-of-1 trial	Randomized trial or (exceptionally) observational study with dramatic effect			

Source: https://www.cebm.ox.ac.uk/resources/levels-of-evidence/ocebm-levels-of-evidence

^a^ Level may be graded down based on study quality, imprecision, indirectness (study PICO does not match questions PICO), because of inconsistency between studies, or because the absolute effect size is very small; level may be graded up if there is a large or very large effect size.

^b^ As always, a systematic review is generally better than an individual study.

#### 2.2.3 Overall judgment of causality

The overall judgment of causality for a particular safety signal should consider the totality of evidence from both individual and aggregate levels before confirming or refuting the signal. Figure 1 suggests a qualitative guide for the contribution of various streams of evidence to the final decision. The relative weights proposed in the figure reflect a suggestion for the strength of the individual sources of evidence based on what is known about the scientific basis of each source. It is intended to illustrate that not all evidence should be treated equally in the overall judgment. How much weight to put on each source of evidence would eventually be left to the assessor of the safety signal. The assessor can vary the weight for each source based on the quality and completeness of the supporting data.

The elements and color codings listed in Figure 1 are tailored to the perceived value of various streams of evidence under three different scenarios: if the evidence is consistent with causality, if it opposes causality, and if it is neutral, absent, or inconclusive. Because of the nature of missing information, especially in the post-marketing setting, the weighting schema is intended to alert the assessor to how to handle the effect of the lack of information. The mere absence of information on a particular element should not always be taken for or against the presence of an association between a drug and an AE.

The use of color coding is intentional as the tool is meant to be used introspectively and qualitatively based on medical and scientific judgment as well as the context of the intricacies around the safety signal regarding the nature of the patient population, the drug, and the AE being investigated. We do not recommend numeric allocation and overall scoring or ranking at this time. Score-based algorithms are often criticized as they assume a linear relationship between various streams of evidence and the eventual judgment on causality, which might not always be true in all scenarios.

## 3 Case Studies

### 3.1 Case study of thrombotic thrombocytopenic purpura and Drug X

Certain rare events are only observed in post-marketing settings after many patients are exposed. In one situation, an event of acquired TTP was observed after exposure of ∼30,000 patients to Drug X, with no cases reported during clinical trials and no non-clinical supportive evidence. Drug X is known to cause autoimmunity and, therefore, a biologically plausible mechanism with a plausible time to onset of 6–12 months was identified. A class effect for this AE was also noted. TTP is a serious condition and presents a diagnostic challenge, especially in the patient population treated with Drug X. This drug is known to cause autoimmune conditions such as ITP, which may have overlapping clinical presentations.

A case definition utilizing clinical guidelines was used to facilitate designing the safety database search strategy and distinguish the ITP cases from TTP cases. The measurement of von Willebrand factor-cleaving protease (ADAMTS13) activity was found to help identify cases of TTP with greater certainty. According to Barrows and Teruya 2014, “Reduced ADAMTS13 activity assay (<20% normal activity) has an overall sensitivity and specificity of 100% and 99%, respectively. The positive predictive value was 91%, and the negative predictive value was 100%.”

The search criteria cast a broad net and retrieved 18 unique case reports. The case definition and the validated diagnostic approach (Barrow and Teruya, 2014) were applied to these case reports, identifying five diagnostically confirmed case reports. No confounders were reported, and time to onset was plausible in four of the five cases. No positive rechallenges or case clusters were detected.

From the literature (NORD)^12^, the overall TTP incidence rate is 4:100,000 people. In the safety dataset obtained for this case study, the observed reporting rates of TTP were 66.05:100,000 (considering all 18 case reports) and 18.34:100,000 (considering only the five diagnostically confirmed cases). When compared to the overall incidence rate (4:100,000), these observed reporting rates were 16.5 and 4.6 times higher than the expected rate of TTP, respectively. No factors for potential stimulated reporting of these cases were evident for this drug.

A probability calculation was made to determine if these cases might be encountered by chance due to the background incidence of TTP in this population. Given the background rate of TTP (4:100,000 per person) and the extent of exposure to Drug X, the probability of observing five cases or more of TTP was 0.005, and the probability of observing 18 or more cases of TTP was 0.000001.

### 3.2 Case study of adult-onset Still’s disease and Drug Y

In this situation, a case definition utilizing clinical guidelines, therapeutic interventions, clinical course, and signs and symptoms was used to facilitate designing the safety database search strategy.

The search criteria cast a broad net and retrieved 13 unique case reports. Applying the case definition and validated Yamaguchi diagnostic criteria confirmed 11 of those case reports. No confounders were reported, and time to onset was plausible in five of the eleven cases. No positive rechallenges or case clusters were detected.

From the literature (Giacomelli et al., 2018), the overall AOSD incidence rate is 0.16–0.40:100,000 per person. In the safety dataset obtained for this case study, the observed reporting rates of AOSD were 39.4:100,000 (considering all 11 case reports) and 17.9:100,000 (considering only the five diagnostically confirmed cases). When compared to the overall incidence rate, these observed reporting rates were 99 and 45 times higher than the expected rate of AOSD, respectively. No factors for potential stimulated reporting of these cases were evident for this drug.

A probability calculation was made to determine whether these cases might be encountered by chance due to the background incidence of AOSD in this population. Given the background rate of AOSD (0.14–0.40 per 100,000 people) and the extent of exposure to Drug Y, the probability of observing five cases or more of AOSD was 0.000001.

The examples presented previously demonstrate the application of many aspects of the proposed approach that strengthened the signal identification and adjudication process. First, a robust case definition and diagnostic criteria were used. In addition to examining the facts suggesting the causality in individual cases, the reporting rates were much higher than the expected background rate, and the probability of observing the reported number of cases due to background rate was near zero, which strengthened the association between the events (TTP and AOSD) and the drugs. The adverse events were confirmed as risks and classified as identified risks.

## 4 Conclusion

Causality assessment for safety signals is challenged by variations and inconsistencies across industries in AE reporting methodology as well as in the interpretation of these reports and the lack of a universally accepted method for determining causality. While present even in the clinical trial setting (Hammad et al., 2011), these challenges are increasingly pronounced in the post-marketing setting, particularly in the context of rare AEs.

The approach proposed in this article utilizes some of the same elements as other established methods of causality assessment but additionally focuses on simplicity and highlights the need for appropriate levels of flexibility, consistency, and transparency in the assessment process. This approach has been created in order to provide safety assessors with a qualitative, structured, consistent approach to the assessment of causality. Consideration is given to 1) characterizing the diagnosis of the clinical condition, 2) assessing the evidence for causality (both at the individual/patient level and the aggregate/population level), and 3) providing specific guidance for weighting various streams of evidence in the decision-making process.

In general, assessing causality in safety signals will always require a level of subjectivity and, thus, will never be an exact science. However, a holistic framework consisting of broadened thought processes and tools is of the utmost importance to approaching causality assessment in a structured manner, which will eventually benefit patients by increasing the accurate identification of true safety issues. This framework could be an asset when facing the upcoming challenges caused by the breakthrough of immunologic and biologic products displaying a new range of adverse reactions. Further research is warranted to validate the performance of the proposed approach in real-life safety signal causality assessment scenarios.

## Data Availability

The original contributions presented in the study are included in the article/Supplementary Material; further inquiries can be directed to the corresponding author.

## Footnotes

1
https://cioms.ch/wp-content/uploads/2018/03/WG8-Signal-Detection.pdf

2
https://database.ich.org/sites/default/files/E2A_Guideline.pdf

3
https://www.ema.europa.eu/en/documents/scientific-guideline/guideline-good-pharmacovigilance-practices-module-vi-management-reporting-adverse-reactions_en-0.pdf

4
https://www.appliedclinicaltrialsonline.com/view/interexpert-agreement-adverse-events-evaluation

5
Thrombotic Thrombocytopenic Purpura (TTP): Practice Essentials, Background, Pathophysiology (medscape.com)

6
https://who-umc.org/signal-work/clinical-assessment/#:∼:text=The%20WHO-UMC%20system%20for%20causality%20assessment%20of%20single,the%20quality%20of%20the%20documentation%20of%20the%20observation

7
https://www.ncbi.nlm.nih.gov/books/NBK570326/

8
https://www.fda.gov/media/150356/download

9
https://stattrek.com/online-calculator/binomial.aspx

10
https://stattrek.com/online-calculator/poisson.aspx

11
https://www.cebm.ox.ac.uk/resources/levels-of-evidence/ocebm-levels-of-evidence

12 National Organization of Rare Diseases (NORD) website. Accessed 2 May 2023, from https://rarediseases.org/rare-diseases/thrombotic-thrombocytopenic-purpura/?filter=Affected+Populations
